# Discordant and inappropriate discordant recommendations in consensus and evidence based guidelines: empirical analysis

**DOI:** 10.1136/bmj-2021-066045

**Published:** 2021-11-25

**Authors:** Liang Yao, Muhammad Muneeb Ahmed, Gordon H Guyatt, Peijing Yan, Xu Hui, Qi Wang, Kehu Yang, Jinhui Tian, Benjamin Djulbegovic

**Affiliations:** 1 Department of Health Research Methods, Evidence, and Impact, McMaster University, Hamilton, ON, Canada; 2 Michael G DeGroote School of Medicine, McMaster University, Hamilton, ON, Canada; 3 Department of Medicine, McMaster University, Hamilton, ON, Canada; 4 Department of Epidemiology and Health Statistics, West China School of Public Health and West China Fourth Hospital, Sichuan University, Chengdu, Sichuan, China; 5 Evidence Based Medicine Centre, Lanzhou University, Lanzhou, Gansu, China; 6 Beckman Research Institute, Department of Computational and Quantitative Medicine, City of Hope, Duarte, CA, USA

## Abstract

**Objective:**

To investigate whether alignment of strength of recommendations with quality of evidence differs in consensus based versus evidence based guidelines.

**Design:**

Empirical analysis.

**Data source:**

Guidelines developed by the American College of Cardiology and the American Heart Association (ACC/AHA) and the American Society of Clinical Oncology (ASCO) up to 27 March 2021.

**Study selection:**

Recommendations were clearly categorised as consensus or evidence based, were separated from the remainder of the text, and included both the quality of evidence and the strength of the recommendations.

**Data extraction:**

Paired authors independently extracted the recommendation characteristics, including type of recommendation (consensus or evidence based), grading system used for developing recommendations, strength of the recommendation, and quality of evidence. The study team also calculated the number of discordant recommendations (strong recommendations with low quality evidence) and inappropriate discordant recommendations (those that did not meet grading of recommendations assessment, development, and evaluation criteria of appropriateness).

**Results:**

The study included 12 ACC/AHA guidelines that generated 1434 recommendations and 69 ASCO guidelines that generated 1094 recommendations. Of the 504 ACC/AHA recommendations based on low quality evidence, 200 (40%) proved to be consensus based versus 304 (60%) evidence based; of the 404 ASCO recommendations based on low quality evidence, 292 (72%) were consensus based versus 112 (28%) that were evidence based. In both ACC/AHA and ASCO guidelines, the consensus approach yielded more discordant recommendations (ACC/AHA: odds ratio 2.1, 95% confidence interval 1.5 to 3.1; ASCO: 2.9, 1.1 to 7.8) and inappropriate discordant recommendations (ACC/AHA: 2.6, 1.7 to 3.7; ASCO: 5.1, 1.6 to 16.0) than the evidence based approach.

**Conclusion:**

Consensus based guidelines produce more recommendations violating the evidence based medicine principles than evidence based guidelines. Ensuring appropriate alignment of quality of evidence with the strength of recommendations is key to the development of “trustworthy” guidelines.

## Introduction

Development of trustworthy guidelines—a collection of related recommendations for clinical practice—is key to improving physicians’ decision making and patients’ outcomes.^1^ Necessary requirements for trustworthiness include assessing quality/certainty of evidence and issuing recommendations graded by strength (strong or weak/conditional). When they make recommendations, many organisations, including the American College of Cardiology and the American Heart Association (ACC/AHA), and the American Society of Clinical Oncology (ASCO), categorise their guidelines as evidence based or consensus based. This practice remains common: authors of numerous recent covid-19 guidelines classify them as consensus based versus evidence based.^2^ ^3^ ^4^ ^5^ ^6^

Organisations adopt different grading systems to rate the quality of evidence and the strength of recommendations, of which GRADE (grading of recommendations assessment, development, and evaluation)^7^ ^8^ is one choice. ACC/AHA and ASCO each use their own systems to develop practice guidelines (appendixes 1 and 2). Guidelines that are labelled as evidence based often include considerable evidence of high or moderate quality and commit to recommendations consistent with underlying evidence—that is, respecting a close link between quality of evidence and strength of recommendations. Consensus based guidelines typically rely on evidence of lower quality, and might not respect the link between quality and strength of recommendations.^9^ ^10^

The failure to ensure concordance between quality of evidence and strength of recommendations violates a key principle of evidence based medicine and risks misleading guidance.^11^ ^12^ ^13^ ^14^ The GRADE working group has identified exceptions to this rule^15^: five characteristic situations in which strong recommendations based on low quality evidence might be appropriate (box 1).

Box 1 **Five paradigmatic situations warranting strong recommendation despite low or very low quality evidence in effect estimates**

**Life threatening or catastrophic clinical situations, potential benefits, low quality evidence**

Example: In patients with life threatening disseminated blastomycosis, use of amphotericin which is more toxic (high quality evidence), but might reduce mortality (low quality evidence), in comparison with itraconazole. After considering the life threatening situation, the guideline panel made a strong recommendation to support the use of amphotericin.^16^

**Uncertain benefit, high certainty of harm**

Example: Patients requiring surgery for symptomatic cataract can undergo either retrobulbar anaesthesia or topical anaesthesia. Retrobulbar anaesthesia might offer some uncertain benefit over topical anaesthesia, supported with low quality evidence (eg, decreased risk of intraoperative zonule tear, iris prolapse, and surgical pain). Moderate to high certainty evidence suggests, however, that retrobulbar anaesthesia is associated with substantial harm, including chemosis, periorbital haematoma and even, severe, life threatening complications. Because the harms are judged to outweigh benefits, the guideline committee issued a strong recommendation against retrobulbar anaesthesia rather than topical anaesthesia for patients undergoing cataract surgery.^17^

**Equivalence for benefits, low quality evidence, one option clearly less risky or costly**

Example: Extranodal marginal zone lymphoma of mucosa associated lymphoid tissue ((MALT lymphoma) is linked to infection with *Helicobacter pylori* bacteria, but historically, MALT lymphoma has been treated with radiation or gastrectomy. Low quality of evidence shows similar response (benefits) with antibiotics for *H pylori* but clearly less harm, morbidity, and cost (high quality evidence). Based on the assessment that benefits of alternative treatments are similar, but the harms associated with antibiotics are plainly lower than those from gastrectomy or radiation, the guideline authors issued a strong recommendation in favour of eradication of *H pylori* bacteria with antibiotics rather than radiation therapy or gastrectomy in patients with MALT lymphoma.^18^

**High certainty in similar benefits, one option potentially more risky or costly**

Example: Whether patients with subclinical hypothyroidism benefit from thyroid hormones more than if they are left untreated, is uncertain. With or without treatment patient have similar benefits for quality of life or thyroid related symptoms, including depressive symptoms, fatigue, and body mass index (moderate to high quality evidence). Low quality evidence suggests, however, that the administration of thyroid hormones is not harmless. In view of this, the guideline panel issued a strong recommendation against prescribing thyroid hormones for adults with subclinical hypothyroidism.^19^

**Uncertain benefits, potential catastrophic harm**

Example: Oral anticoagulants and antiplatelet agents are often prescribed for patients with chronic coronary or other arterial diseases. Low quality evidence shows that the combination of oral anticoagulants and antiplatelet agents is associated with a high risk of serious bleeding. Hence the European Society of Cardiology made a strong recommendation against the use of a combination of oral anticoagulant and antiplatelet agents in patients with chronic coronary or other arterial disease and in favour of treatment with a single oral anticoagulant.^20^

In their approach to particular guidelines, some organisations, including ACC/AHA and ASCO, classify their guidelines as evidence based when much of the supporting evidence is deemed moderate or high quality. When most of the evidence is of low or very low quality, these organisations then often categorise their approach as consensus based.^21^ ^22^ ^23^ In doing so, organisations seem to characterise consensus based recommendations as highly reliant on expert opinion, but this might not be the case for recommendations that are considered evidence based.

Both consensus and evidence based approaches require judicious consideration of the relevant evidence, expert interpretation of the evidence, and ultimately. panel consensus. In other words, guideline panels must always carefully consider the available evidence, regardless of quality, and must always rely on expert insights to arrive at consensus recommendations^14^; in this sense, denoting evidence based and consensus based recommendations as separate categories is misleading.^10^ Intention-to-treat consensus based guidelines as a separate category of guidelines seem to purposefully relax the necessary requirement that the strength of the recommendations should align with the underlying quality of evidence, which in turn, might result in inappropriate discordant recommendations.

If this is the case, one would expect a greater number of discordant recommendations, particularly inappropriate discordant recommendations, in consensus based guidelines than in evidence based guidelines. To date, however, empirical support for this expected finding^10^ is lacking. We provide an empirical assessment of how often consensus versus evidence based guidelines issued strong recommendations based on low quality evidence (discordant) and how many of them are inappropriate (inappropriate discordant recommendations). Because these are the world’s leading professional organisations developing recommendations for people with cardiovascular diseases and cancer, the leading cause of morbidity and mortality in much of the world, we evaluated ACC/AHA and ASCO guidelines. Recommendations by these organisations affect the decisions of thousands of physicians and outcomes for millions of patients worldwide.

## Methods

### Guideline source and inclusion, and data extraction

We searched the ACC/AHA (https://www.acc.org/guidelines#/doctype=Guidelines) and ASCO websites (https://www.asco.org/) on 5 May 2020 and updated the search on 27 March, 2021. Eligible guidelines adhered to the following criteria: (a) explicitly distinguished consensus from an evidence based approach to the development of guidelines; (b) included recommendations; (c) clearly categorised the recommendations as consensus versus evidence based and separated them from the remainder of the text; and (d) included both the quality of evidence and the strength of each recommendation. When several versions of the guidelines existed, we used the most recent.

Paired reviewers independently extracted the characteristics of eligible guidelines, including the title, year, version, recommendations, grading system, quality of evidence for each recommendation, and the strength of recommendations.

### Quality of evidence and strength of recommendations

ACC/AHA guidelines categorise the quality of evidence as high, moderate, and low, and the strength of recommendations as strong and weak (appendix 1).^21^ ASCO guidelines categorise the quality of evidence as high, moderate (intermediate), low, and insufficient, and the strength of recommendations as strong, moderate, and weak (appendix 2).^22^ ^23^ Although the two organisations use different systems to rate the quality of evidence and strength of recommendations, they provide similar definitions of the two concepts. For example, strong recommendations in both ACC/AHA and ASCO guidelines indicate no concerns about the recommendation reflecting the best practice. Similarly, low quality evidence in the ACC/AHA guidelines corresponds to low and insufficient quality evidence in the ASCO guidelines. To enable comparison across organisations, we combined low and insufficient quality evidence in the ASCO guidelines and labelled them as low quality.

### Characterising the type of recommendations

Both ACC/AHA and ASCO develop guidelines according to the following principle: when evidence is sufficient, the panels are instructed to use the evidence based approach to inform recommendations; when evidence is insufficient, the panels use a consensus based approach.^21^ ^22^ In some instances, even when the quality of evidence is moderate or high, if the ASCO guideline panel members have a high level of agreement about the benefits and harms of the intervention, they might also use the consensus approach to assess the quality of evidence and issue recommendations. ACC/AHA guidelines denoted consensus based recommendations as guidelines based on experts’ opinion; ASCO guidelines categorised each recommendation as consensus versus evidence based (see box 2 and box 3 for examples of consensus based and evidence based recommendations in ACC/AHA and ASCO guidelines, respectively).

Box 2 **Examples of consensus based and evidence based recommendations in ACC/AHA guidelines^24^**

**Consensus based recommendation**

- Question: For patients with valvular heart disease for whom intervention is contemplated, should individual risks be assessed?
- Recommendation: For patients with valvular heart disease for whom intervention is contemplated, individual risks should be calculated for specific surgical or transcatheter procedures, using online tools when available, and discussed before the procedure as part of a shared decision making process.
- (Type: expert opinion (consensus based); evidence quality: level C (low quality); strength of recommendation: 1 (strong))

**Evidence based recommendation**

- Question: In patients aged >65 years who require aortic valve replacement, should a bioprosthesis or a mechanical valve be offered?
- Recommendation: In patients aged >65 years who require aortic valve replacement, it is reasonable to choose a bioprosthesis over a mechanical valve.
- (Type: evidence based; evidence quality: level B (moderate); strength of recommendation: 2a (moderate))

Box 3 **Examples of consensus based and evidence based recommendations in ASCO guidelines 25**

**Consensus based recommendation**

- Question: In treatment of cancer related anaemia, what special considerations apply to adult patients with non-myeloid haematologic malignancies who are receiving concurrent myelosuppressive chemotherapy?
- Recommendation: In patients with myeloma, non-Hodgkin lymphoma, or chronic lymphocytic leukaemia, clinicians should observe the haematologic response to cancer treatment before considering an erythropoiesis stimulating agent.
- (Type: informal consensus; evidence quality: low; strength of recommendation: moderate)

**Evidence based recommendation**

- Question: To reduce the need for red blood cell transfusions, should erythropoiesis stimulating agents be offered to patients who have chemotherapy associated anaemia?
- Recommendation: Depending on clinical circumstances, erythropoiesis stimulating agents can be offered to patients with chemotherapy associated anaemia whose cancer treatment is not curative in intent and whose haemoglobin has declined to 100 g/L. Transfusion of red blood cells is also an option, depending on the severity of the anaemia or clinical circumstances.
- (Type: evidence based; evidence quality: high; strength of recommendation: strong)

### Characterising the appropriateness of recommendations

For recommendations with low quality evidence, two reviewers (LY and MMA) independently assessed whether or not discordant recommendations were or were not inappropriate (that is, whether they met GRADE criteria for appropriate discordant recommendations; see examples in box 1 for appropriate discordant recommendations and the first example in box 2 for inappropriate discordant recommendations).^15^ Because judgement of the appropriateness of discordant recommendations is potentially the most challenging, we calculated the chance corrected agreement (κ statistics) between reviewers for this outcome.

### Data analysis

We summarised the number, type, and composition of recommendations and measured the discrepancies between consensus based and evidence based guidelines: strong recommendation based on low quality (discordant recommendation), and inappropriate discordant recommendation. Because recommendations (lower level units) were nested in guidelines (higher level units), we used multilevel mixed effect logistic regression,^26^ ^27^ ^28^ in which the responses were clustered within guideline panels. We estimated discrepancies between consensus based and evidence based guidelines by odds ratios. In this modelling convention, an odds ratio greater than 1.0 suggests that consensus based guidelines issued more discordant or inappropriate discordant recommendations than evidence based guidelines. In addition, we summarised the proportion of recommendations “against” versus “in favour of” interventions among the discordant recommendations and inappropriate discordant recommendations. For all statistical analyses, all P values were two sided. We performed all analyses using Stata (College Station, TX) version 15 software.

### Patient and public involvement

Owing to the nature of this work, we did not include patients and public.

## Results

### Guideline characteristics

The search yielded 12 eligible ACC/AHA guidelines, published between 2013 and 2021 (appendix 3), with the median number of recommendations from each guideline 109 (interquartile range (74-139), totalling 1434 recommendations. Among the ASCO guidelines, 69 published between 2012 and 2021 (appendix 3) met the inclusion criteria; the median number of recommendations was 14 (interquartile range 3-34), with a total of 1094 recommendations. Table 1 and table 2 present the quality of evidence and strength of recommendations in the guidelines.

**Table 1:** Quality of evidence and strength of recommendations in consensus and evidence based recommendations in American College of Cardiology/American Heart Association guidelines

Strength of recommendations	Quality of evidence		
	High	Moderate	Low
Consensus approach (n=200):			
Strong	0	0	115 (58)
Moderate	0	0	50 (25)
Weak	0	0	35 (18)
Totals (%)	0	0	200 (100)
Evidence approach (n=1234):			
Strong	81 (83)	402 (48)	117 (38)
Moderate	15 (15)	311 (37)	118 (39)
Weak	2 (2)	119 (14)	69 (23)
Totals (%)	98 (100)	832 (100)	304 (100)

**Table 2:** Quality of evidence and strength of recommendations in consensus and evidence based recommendations in American Society of Clinical Oncology guidelines

Strength of recommendations	Quality of evidence		
	High	Moderate	Low
Consensus approach (n=358):			
Strong	4 (100)	18 (29)	92 (32)
Moderate	0	37 (60)	141(48)
Weak	0	7 (11)	59 (20)
Totals (%)	4 (100)	62 (100)	292 (100)
Evidence approach (n=736):			
Strong	225 (89)	162 (44)	30 (27)
Moderate	26 (10)	189 (51)	39 (35)
Weak	3 (1)	19 (5)	43 (38)
Totals (%)	254 (100)	370 (100)	112 (100)

ACC/AHA guidelines included 504 recommendations with low quality evidence, of which 200 (40%) were consensus based and 304 (60%) were evidence based; for the ASCO guidelines, 404 recommendations were based on low quality evidence, of which 292 (72%) were based on consensus approach and 112 (28%) were evidence based (table 3). Appendix 4 presents more details of included guidelines.

**Table 3:** Appropriateness of recommendations with low quality evidence

Appropriateness	Type of recommendations	
	Consensus approach	Evidence approach
ACC/AHA (n=504):		
Appropriate	89 (45)	209 (69)
Inappropriate	111 (56)	95 (31)
Totals (%)	200 (100)	304 (100)
ASCO (n=404):		
Appropriate	220 (75)	102 (91)
Inappropriate	72 (25)	10 (9)
Totals (%)	292 (100)	112 (100)

ACC/AHA=American College of Cardiology and the American Heart Association; ASCO=American Society of Clinical Oncology.

### Strong recommendations based on low quality evidence (discordant recommendations)

**Fig 1:**
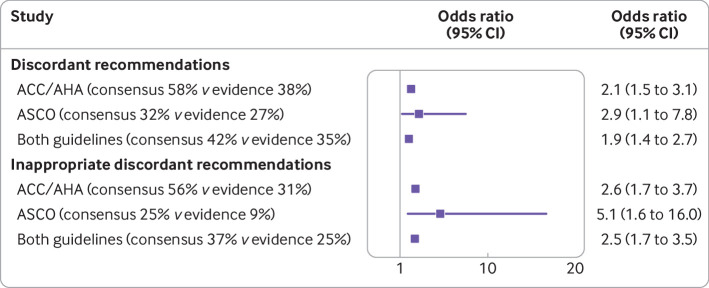
The proportion of discordant recommendations and inappropriate discordant recommendations in consensus versus evidence based methods of guidelines development. The odds ratio (95% confidence interval) estimates were generated from the multilevel model (appendix 4). Odds ratio >1 indicates that guidelines developed by consensus based methods generate more discordant or inappropriate discordant recommendations than the guidelines that employ evidence based approaches. ACC/AHA=American College of Cardiology and the American Heart Association; ASCO=American Society of Clinical Oncology

Of recommendations based on low quality evidence, using the consensus based approach ACC/AHA issued strong recommendations for 58% (n=115) versus 38% (n=117) using the evidence based approach (odds ratio 2.1, 95% confidence interval 1.5 to 3.1, appendix 5 and fig 1). Thus guidelines using a consensus based approach, on average, have 2.1 times greater odds of issuing discordant recommendations than those employing evidence based methodology. We obtained a similar result in the analysis of ASCO guidelines: of recommendations based on low quality evidence, 32% (n=92) proved discordant by a consensus based approach versus 27% (n=30) by evidence based methods (odds ratio 2.9, 95% confidence interval 1.1 to 7.8, appendix 5 and fig 1). The odds ratio of consensus based versus evidence based approach for ACC/AHA and ASCO guidelines combined was 1.9 (1.4 to 2.7).

Consensus based guidelines generated more discordant recommendations in favour of, than against, health interventions (appendix 5). In ACC/AHA guidelines, 98% (n=113) versus 2% (n=2) discordant recommendations were in favour of, than against, interventions using the consensus method, but when relying on an evidence based approach, the proportions of discordant recommendations were 81% (n=95) versus 19% (n=22). ASCO guidelines generated 83% (n=76) versus 17% (n=16) discordant recommendations in favour of versus against interventions when using consensus based methods, but when using evidence based methods, the proportions of discordant recommendations were 60% (n=18) versus 40% (n=12; appendix 5).

### Inappropriate discordant recommendations

We identified 26 discordant recommendations in ACC/AHA guidelines and 40 in ASCO guidelines that met the five characteristic situations in which issuing discordant recommendations might be justified (κ statistic for agreement between the reviewers was 0.74 for ACC/AHA guidelines and 0.81 for ASCO guidelines). The remaining 206 discordant recommendations in ACC/AHA guidelines and 82 in ASCO guidelines represent inappropriate discordant recommendations (table 3). The consensus based approach generated 2.6 times higher odds of more inappropriate discordant recommendations over evidence based guidelines in ACC/AHA guidelines (56% *v* 31%; odds ratio 2.6, 95% confidence interval 1.7 to 3.7, appendix 5 and fig 1) and 5.1 times greater odds of inappropriate discordant recommendations in ASCO guidelines (25% *v* 9%; 5.1, 1.6 to 16.0, appendix 5 and fig 1). The odds ratio of consensus based versus evidence based approach for ACC/AHA and ASCO guidelines combined was 2.5, 1.7 to 3.5 (37% *v* 25%, appendix 5 and fig 1).

ASCO guidelines generated more inappropriate discordant recommendations in favour of than against interventions using consensus based (79% *v* 21%) rather than evidence based methods (50% *v* 50%). In ACC/AHA guidelines, almost all consensus and evidence based recommendations were in favour of recommended interventions (appendix 6).

## Discussion

### Principal findings

This study reviewed 1434 ACC/AHA recommendations and 1094 ASCO recommendations and found that consensus based guidelines, in the face of low quality evidence, have 1.9 times greater odds of issuing strong recommendations (discordant recommendations)—with 2.1 times greater odds in ACC/AHA guidelines, and 2.9 times greater odds in ASCO guidelines—and 2.5 times greater odds of issuing inappropriate discordant recommendations than guidelines that were categorised as evidence based—with 2.6 times greater odds in ACC/AHA guidelines, and 5.1 times greater odds in ASCO guidelines. An additional important finding was the 31% frequency of discordant recommendations in the evidence based ACC/AHA guidelines (table 3).

### Strength and limitations

Strengths of our study include rigour of data abstraction and analysis. In particular, we used multilevel modelling to obtain an accurate effect when the data were nested.^29^ ^30^ This method proved important: results showed clustering in each guideline—particularly in the ASCO guidelines—for the effect on discordant and inappropriate recommendations. This study provides empirical evidence for the difference between recommendations informed by consensus versus evidence based approaches. We analysed guidelines developed by two leading organisations (ACC/AHA and ASCO), which deal with the leading causes of morbidity and mortality worldwide. The methods used by these organisations are likely to reflect those of smaller professional organisations and are, therefore, probably generalisable. Although other organisations have a less clear definition of consensus versus evidence based guidelines, many rely on a similar distinction between the two methods for guidelines development, including the Society of Thoracic Surgeons, European Association of Urology, and Society for Immunotherapy of Cancer.

One limitation of our study is that we did not make our own detailed assessment of evidence quality to verify the authors’ rating of quality of evidence. Thus it remains possible that some of the discordant recommendations were not truly discordant—that is, that evidence of moderate or high quality was misclassified as low quality by the authors. Nevertheless, the guidelines panels made their strong recommendations with the understanding that underlying quality evidence was low. Other limitations are related to the use by ACC/AHA and ASCO guidelines of different grading systems to rate the quality of evidence and strength of recommendations, which creates problems in comparing results. Nonetheless, both organisations clearly distinguish the quality (certainty) of evidence from the strength of recommendations. Although reproducibility of rating of quality of evidence might differ between these two organisations, it is clear that when issuing their expert/consensus based recommendations, both organisations have instructed their panels to decouple quality of evidence from strength of recommendations.

Some recommendations might have been classified as good practice statements (as characterised by GRADE).^31^ In good practice statements, a large and compelling body of indirect evidence strongly supports the recommended action, but the statements are not formally graded recommendations.^31^ We did not specifically look for good practice statements among the discordant recommendations. All recommendations we reviewed, however, were based on an assessment of the quality of evidence and strength of recommendations.

In this paper, we focus on strong recommendations based on low quality evidence. Analysis of weak recommendations based on high quality of evidence was beyond the scope of this study, but within the GRADE system would be appropriate whenever high quality evidence showed a close balance between desirable consequences of an intervention and a comparator. In such situations, when net benefit or harm is minimal, differences in patients’ values and preferences would lead to fully informed patients making different choices, and thus appropriate weak recommendations.

ASCO guidelines generated a relatively small number of 10 (9%) inappropriate discordant recommendations. The multilevel analysis limits the power of the analysis. These two problems resulted in very wide 95% confidence intervals around the ASCO outcomes. A small number of discordant inappropriate recommendations also prevented full scale multilevel analysis; as a result, we reported only descriptive analyses for against versus in favour of intervention. This study was conducted because insights obtained from preliminary analyses informed the more sophisticated and detailed analyses that followed; therefore, the study was not preregistered as the protocol.

### Comparison with prior studies

Three previous studies by three different organisations identified discordant recommendations and calculated how many were inappropriate. A study of World Health Organization guidelines reported that of 302 recommendations supported by low or very low quality evidence, 160 (53%) were discordant and 73 (24%) were inappropriate.^32^ In 256 Endocrine Society recommendations supported by low or very low quality evidence, panels made strong recommendations in 121 (47%), and reviewers judged 33 (13%) as inappropriate.^33^ In contrast, of 4335 recommendations based on low quality evidence in UpToDate (an electronic clinical resource tool for physicians and patients), 366 (8.4%) were discordant and 145 (3.3%) proved inappropriate.^34^

Because WHO, the Endocrine Society, and UpToDate issue only evidence based recommendations, the appropriate comparators in our study are evidence based recommendations from ASCO and ACC/AHA. Thus, the performance of ASCO for evidence based recommendations (9% inappropriate) falls between UpToDate and the other two organisations. In comparison, 31% of ACC/AHA inappropriate discordant recommendations were evidence based.

No previous study has compared discordant recommendations in evidence based versus consensus based guidelines.

### Implications

Two leading professional organisations issue guidelines that they label as “consensus”—and recommendations categorised as “evidence based”—that include many inappropriate discordant recommendations, which raises serious concerns.^11^ Guideline panels should be considerably more inclined to issue strong recommendations when the quality of evidence is high than when evidence is of low or very low quality. Indeed, as the essential condition of strong recommendations—that is, the benefits clearly outweigh harms and burdens—will seldom be fulfilled, strong recommendations based on low or very low quality evidence are seldom appropriate.^11^ ^35^

It is further problematic that the guideline development organisations seem to have a deliberate policy of making strong recommendations when evidence is only of low quality. According to the ACC/AHA and ASCO guideline methodology,^21^ ^22^ ^23^ when the evidence is of sufficient quality, the guideline panel should use an evidence based approach to inform recommendations. Otherwise, the panel should rely on consensus or expert driven recommendations. Classifying guidelines as consensus based seems to provide a licence to panels to be less disciplined in ensuring that the strength of recommendations is consistent with the underlying quality of evidence.

In doing so, these organisations run the risk of patients receiving treatment which, with shared decision making and acknowledging the uncertainty of evidence, they would not have otherwise chosen. The reason for this risk is that strong recommendations are intended as “just do it” guidance, in which a panel has concluded that all, or almost all, fully informed patients would make the same choice. These recommendations are therefore intended to help physicians, all of whom are time constrained, take the time for shared decision making when it is most important—including when low quality evidence provides a decision by considering the patient’s value and preference. Inappropriate strong recommendations might have other problematic consequences, including constraining future randomised trials that would generate higher quality evidence.

Our results also show that classifying a guideline as evidence based provides only limited protection against inappropriate discordant recommendations. These occurred in 9% of ASCO evidence based guidelines informed by low quality evidence and 31% of corresponding ACC/AHA evidence based guidelines. This finding might indicate a conscious alternative perspective on guideline recommendations: those strong recommendations are appropriate even when one is uncertain about benefits and harms. Alternatively, it might indicate persistence of a tradition of strong recommendations based on the convictions of expert panellists.

### Conclusion

The consensus methodology employed by ACC/AHA and ASCO guidelines was associated with inappropriate discordant recommendations, which might lead to patients receiving interventions for which the benefits do not clearly outweigh the harms, and that might be inconsistent with their values and preferences. Our results support re-examining the wisdom of developing consensus based guidelines, and promoting enhanced rigour to ensure, whatever label the recommendations are given, appropriate alignment of quality of evidence with strength of recommendations.

**What is already known on this topic**

Many organisations explicitly classify their guidelines as evidence based when much of the supporting evidence is deemed moderate or high quality; when most of the evidence is of low quality or very low quality, these organisations often categorise their approach as expert or consensus based
A central principle of evidence based medicine is that the strength of recommendations should be consistent with the underlying quality of evidence
It is not clear if consensus based guidelines violate this key evidence based medicine principle—that is, whether alignment of strength of recommendations with quality of evidence differs in consensus based versus evidence based guidelines

**What this study adds**

Consensus based guidelines generated more inappropriate strong recommendations than evidence based guidelines
For both evidence based and consensus based guidelines, it is important to ensure appropriate alignment of quality of evidence with strength of recommendations
American College of Cardiology/American Heart Association and the American Society of Clinical Oncology—two of the largest world organisations that develop guidelines for heart disease and cancer (leading causes of death), produce overall 41% and 20% inappropriate and probably harmful recommendations based on low quality evidence; when they use consensus rather than evidence based methods they have 2.6 and 5.1 times greater odds of issuing inappropriate discordant recommendations, respectively

## Data Availability

No additional data available.

## Web extra

Extra material supplied by authors

Web appendix: Supplemental appendix

## Notes

Contributors: BD and GHG conceived and designed the research questions. LY and MMA performed the guideline selection. LY, MMA, PY, XH, and JT extracted data. LY, MMA, and KY judged the appropriateness of the recommendations. LY, PY, and BD analysed data. BD, LY, and GHG interpreted the results. LY wrote the manuscript under the supervision of BD and GHG. All authors edited and commented on the manuscript. LY and MMA had full access to all of the data in the study and take responsibility for the integrity of the data and the accuracy of the data analysis. The corresponding author attests that all listed authors meet authorship criteria and that no others meeting the criteria have been omitted.

The lead author (LY) affirms that the manuscript is an honest, accurate, and transparent account of the study being reported; that no important aspects of the study have been omitted; and that any discrepancies from the study as planned (and, if relevant, registered) have been explained.

Dissemination to participants and related patient and public communities: The authors plan to disseminate the paper widely to guideline methodologists, researchers, clinicians, policy makers, and any other associated stakeholders through national and international conferences, webinars, and social media, and by establishing an email discussion group.

Provenance and peer review: Not commissioned; externally peer reviewed.
