# Supplementary material for: Discordant and inappropriate discordant recommendations in consensus and evidence based guidelines: empirical analysis
Source: BMJ. 2021 Nov 25;375:e066045. doi: 10.1136/bmj-2021-066045 (PMC8613613; doi:10.1136/bmj-2021-066045)
Supplement: Supplementary file 1 — Web appendix: Supplemental appendix [file yaol066045.ww.pdf]

Supplemental appendix

Consensus-based guidelines generate more recommendations violating evidence based-medicine principles than evidence-based guidelines: an empirical analysis

Liang Yao, Muhammad Muneeb Ahmed, Gordon H Guyatt, Peijing Yan, Xu Hui, Qi Wang, Kehu Yang, Jinhui Tian, Benjamin Djulbegovic

Table of Contents

Appendix 1. The level of evidence and strength of recommendations in ACC/AHA guidelines . 3

Appendix 2: The level of evidence and strength of recommendations in ASCO guidelines 3

Appendix 3. The year distribution of guidelines and recommendations 5

Appendix 4. Included guidelines and recommendations 5

Appendix 5: Data analysis codes in STATA/SE 15.1 14

*Appendix 6. Proportion of recommendations with "against" vs "favor" interventions in the discordant recommendations and inappropriate discordant recommendations** 16

Appendix 7 : References of included guidelines 16

Appendix 1. The level of evidence and strength of recommendations in ACC/AHA guidelines

CLASS (STRENGTH) OF RECOMMENDATION

CLASS 1 (STRONG) — **Benefit >>> Risk**

Suggested phrases for writing recommendations:
- Is recommended
- Is indicated/useful/effective/beneficial
- Should be performed/administered/other
- Comparative-Effectiveness Phrases†:
  - Treatment/strategy A is recommended/indicated in preference to treatment B
  - Treatment A should be chosen over treatment B

CLASS 2a (MODERATE) — **Benefit >> Risk**

Suggested phrases for writing recommendations:
- Is reasonable
- Can be useful/effective/beneficial
- Comparative-Effectiveness Phrases†:
  - Treatment/strategy A is probably recommended/indicated in preference to treatment B
  - It is reasonable to choose treatment A over treatment B

CLASS 2b (WEAK) — **Benefit ≥ Risk**

Suggested phrases for writing recommendations:
- May/might be reasonable
- May/might be considered
- Usefulness/effectiveness is unknown/unclear/uncertain or not well-established

CLASS 3: No Benefit (MODERATE) — **Benefit = Risk**
(Generally, LOE A or B use only)

Suggested phrases for writing recommendations:
- Is not recommended
- Is not indicated/useful/effective/beneficial
- Should not be performed/administered/other

Class 3: Harm (STRONG) — **Risk > Benefit**

Suggested phrases for writing recommendations:
- Potentially harmful
- Causes harm
- Associated with excess morbidity/mortality
- Should not be performed/administered/other

LEVEL (QUALITY) OF EVIDENCE‡

LEVEL A
- High-quality evidence‡ from more than 1 RCT
- Meta-analyses of high-quality RCTs
- One or more RCTs corroborated by high-quality registry studies

LEVEL B-R — **(Randomized)**
- Moderate-quality evidence‡ from 1 or more RCTs
- Meta-analyses of moderate-quality RCTs

LEVEL B-NR — **(Nonrandomized)**
- Moderate-quality evidence‡ from 1 or more well-designed, well-executed nonrandomized studies, observational studies, or registry studies
- Meta-analyses of such studies

LEVEL C-LD — **(Limited Data)**
- Randomized or nonrandomized observational or registry studies with limitations of design or execution
- Meta-analyses of such studies
- Physiological or mechanistic studies in human subjects

LEVEL C-EO — **(Expert Opinion)**
- Consensus of expert opinion based on clinical experience

COR and LOE are determined independently (any COR may be paired with any LOE).

A recommendation with LOE C does not imply that the recommendation is weak. Many important clinical questions addressed in guidelines do not lend themselves to clinical trials. Although RCTs are unavailable, there may be a very clear clinical consensus that a particular test or therapy is useful or effective.

* The outcome or result of the intervention should be specified (an improved clinical outcome or increased diagnostic accuracy or incremental prognostic information).

† For comparative-effectiveness recommendations (COR 1 and 2a; LOE A and B only), studies that support the use of comparator verbs should involve direct comparisons of the treatments or strategies being evaluated.

‡ The method of assessing quality is evolving, including the application of standardized, widely-used, and preferably validated evidence grading tools; and for systematic reviews, the incorporation of an Evidence Review Committee.

COR indicates Class of Recommendation; EO, expert opinion; LD, limited data; LOE, Level of Evidence; NR, nonrandomized; R, randomized; and RCT, randomized controlled trial.

Appendix 2: The level of evidence and strength of recommendations in ASCO guidelines

Type of Recommendation	Definition
Evidence-based	There was sufficient evidence from published studies to inform a recommendation to guide clinical practice
Formal Consensus	The available evidence was deemed insufficient to inform a recommendation to guide clinical practice. Therefore, the expert Panel used a formal consensus process to reach this recommendation, which is considered the best current guidance for practice. The Panel may choose to provide a rating for the strength of the recommendation (i.e., “strong,” “moderate,” or “weak”). The results of the formal consensus process are summarized in the guideline and reported in an online data supplement.
Informal Consensus	The available evidence was deemed insufficient to inform a recommendation to guide clinical practice. The recommendation is considered the best current guidance for practice, based on informal consensus of the expert Panel. The Panel agreed that a formal consensus process was not necessary for reasons described in the literature review and discussion. The Panel may choose to provide a rating for the strength of the recommendation (i.e., “strong,” “moderate,” or “weak”).

level of evidence	Definition
High	High confidence that the available evidence reflects the true magnitude and direction of the net effect (e.g., balance of benefits versus harms) and further research is very unlikely to change either the magnitude or direction of this net effect.
Intermediate	Intermediate confidence that the available evidence reflects the true magnitude and direction of the net effect. Further research is unlikely to alter the direction of the net effect, however it might alter the magnitude of the net effect.
Low	Low confidence that the available evidence reflects the true magnitude and direction of the net effect. Further research may change the magnitude and/or direction of this net effect.
Insufficient	Evidence is insufficient to discern the true magnitude and direction of the net effect. Further research may better inform the topic. Reliance on consensus opinion of experts may be reasonable to provide guidance on the topic until better evidence is available.
Strength of Recommendation	Definition
Strong	There is high confidence that the recommendation reflects best practice. This is based on: a) strong evidence for a true net effect (e.g., benefits exceed harms); b) consistent results, with no or minor exceptions; c) minor or no concerns about study quality; and/or d) the extent of panelists' agreement. Other compelling considerations (discussed in the guideline's literature review and analyses) may also warrant a strong recommendation
Moderate	There is moderate confidence that the recommendation reflects best practice. This is based on: a) good evidence for a true net effect (e.g., benefits exceed harms); b) consistent results, with minor and/or few exceptions; c) minor and/or few concerns about study quality; and/or d) the extent of panelists' agreement. Other compelling considerations (discussed in the guideline's literature review and analyses) may also warrant a moderate recommendation.
Weak	There is some confidence that the recommendation offers the best current guidance for practice. This is based on: a) limited evidence for a true net effect (e.g., benefits exceed harms); b) consistent results, but with important exceptions; c) concerns about study quality; and/or d) the extent of panelists' agreement. Other considerations (discussed in the guideline's literature review and analyses) may also warrant a weak recommendation.

Appendix 3. The year distribution of guidelines and recommendations

Year	ACC/AHA		ASCO		Combined	
	Number of guidelines	Number of recommendations	Number of guidelines	Number of recommendations	Number of guidelines	Number of recommendations
2012	-	-	1	3	1	3
2013	1	56	0	0	1	56
2014	0	0	2	24	2	24
2015	0	0	2	19	2	19
2016	0	0	8	99	8	99
2017	2	191	13	253	15	444
2018	2	299	15	223	17	522
2019	5	484	12	208	17	692
2020	1	132	12	171	13	303
2021	1	272	4	94	5	366
Overall	12	1434	69	1094	81	2528

Appendix 4. Included guidelines and recommendations

ID	Guideline title	Organization	Year	# of consensus recommendation	# of evidence based recommendations	Consensus approach		Evidence approach	
						# of discordant recommendation	# of inappropriate discordant recommendation	# of discordant recommendation	# of inappropriate discordant recommendation
1	Initial Management of Noncastrate Advanced, Recurrent, or Metastatic Prostate Cancer: ASCO Guideline Update [1]	ASCO	2021	0	15	0	0	1	1
2	Management of Dyspnea in Advanced Cancer: ASCO Guideline [2]	ASCO	2021	0	15	0	0	1	1
3	Neoadjuvant Chemotherapy, Endocrine Therapy, and Targeted Therapy for Breast Cancer: ASCO Guideline [3]	ASCO	2021	9	8	1	1	0	0

4	Therapy for Stage IV Non-Small-Cell Lung Cancer With Driver Alterations: ASCO and OH (CCO) Joint Guideline Update [4]	ASCO	2021	38	9	0	0	0	0
5	Diagnosis and Management of Squamous Cell Carcinoma of Unknown Primary in the Head and Neck: ASCO Guideline [5]	ASCO	2020	1	32	0	0	0	0
6	Estrogen and Progesterone Receptor Testing in Breast Cancer: ASCO/CAP Guideline Update [6]	ASCO	2020	5	8	0	0	0	0
7	Germline and Somatic Tumor Testing in Epithelial Ovarian Cancer: ASCO Guideline [7]	ASCO	2020	1	9	0	0	0	0
8	Lung Cancer Surveillance After Definitive Curative-Intent Therapy: ASCO Guideline [8]	ASCO	2020	7	2	0	0	0	0
9	Management of Cancer Cachexia: ASCO Guideline [9]	ASCO	2020	2	2	0	0	0	0
10	Management of Hereditary Breast Cancer: American Society of Clinical Oncology, American Society for Radiation Oncology, and Society of Surgical Oncology Guideline [10]	ASCO	2020	17	5	0	0	0	0
11	Management of Male Breast Cancer: ASCO Guideline [11]	ASCO	2020	16	0	8	2	0	0
12	Molecular Biomarkers in Localized Prostate Cancer: ASCO Guideline [12]	ASCO	2020	1	6	0	0	0	0
13	Optimum Imaging Strategies for Advanced Prostate Cancer: ASCO Guideline [13]	ASCO	2020	9	5	0	0	0	0
14	Prevention and Management of Chemotherapy-Induced Peripheral Neuropathy in Survivors of Adult Cancers: ASCO Guideline Update [14]	ASCO	2020	0	14	0	0	2	1
15	Systemic Therapy for Melanoma: ASCO Guideline [15]	ASCO	2020	5	6	0	0	0	0
16	Therapy for Stage IV Non-Small-Cell Lung Cancer Without Driver Alterations: ASCO and OH (CCO) Joint Guideline Update [16]	ASCO	2020	0	18	0	0	0	0
17	Adjuvant Therapy for Resected Biliary Tract Cancer: ASCO Clinical Practice Guideline [17]	ASCO	2019	0	2	0	0	0	0

18	Duration of Oxaliplatin-Containing Adjuvant Therapy for Stage III Colon Cancer: ASCO Clinical Practice Guideline [18]	ASCO	2019	1	2	1	1	0	0
19	Management of Cancer-Associated Anemia With Erythropoiesis-Stimulating Agents: ASCO/ASH Clinical Practice Guideline Update [19]	ASCO	2019	7	5	2	1	0	0
20	Management of Osteoporosis in Survivors of Adult Cancers With Nonmetastatic Disease: ASCO Clinical Practice Guideline [20]	ASCO	2019	1	9	1	0	0	0
21	Management of the Neck in Squamous Cell Carcinoma of the Oral Cavity and Oropharynx: ASCO Clinical Practice Guideline [21]	ASCO	2019	0	20	0	0	0	0
22	Medication-Related Osteonecrosis of the Jaw: MASCC/ISOO/ASCO Clinical Practice Guideline [22]	ASCO	2019	11	2	0	0	0	0
23	Potentially Curable Pancreatic Adenocarcinoma: ASCO Clinical Practice Guideline Update [23]	ASCO	2019	7	8	1	1	2	2
24	Role of Patient and Disease Factors in Adjuvant Systemic Therapy Decision Making for Early-Stage, Operable Breast Cancer: Update of the ASCO Endorsement of the Cancer Care Ontario Guideline [24]	ASCO	2019	4	6	1	1	0	0
25	Treatment of Multiple Myeloma: ASCO and CCO Joint Clinical Practice Guideline [25]	ASCO	2019	3	43	0	0	0	0
26	Use of Biomarkers to Guide Decisions on Adjuvant Systemic Therapy for Women With Early-Stage Invasive Breast Cancer: ASCO Clinical Practice Guideline Update—Integration of Results From TAILORx [26]	ASCO	2019	14	20	10	10	0	0
27	Use of Endocrine Therapy for Breast Cancer Risk Reduction: ASCO Clinical Practice Guideline Update [27]	ASCO	2019	0	21	0	0	0	0
28	Venous Thromboembolism Prophylaxis and Treatment in Patients With Cancer: ASCO Clinical Practice Guideline Update [28]	ASCO	2019	8	14	1	1	0	0
29	Antimicrobial Prophylaxis for Adult Patients With Cancer-Related Immunosuppression: ASCO and IDSA Clinical Practice Guideline Update Summary [29]	ASCO	2018	5	5	0	0	0	0

30	Evaluating Susceptibility to Pancreatic Cancer: ASCO Provisional Clinical Opinion [30]	ASCO	2018	10	0	6	2	0	0
31	Hypofractionated Radiation Therapy for Localized Prostate Cancer: An ASTRO, ASCO, and AUA Evidence-Based Guideline [31]	ASCO	2018	0	18	0	0	2	1
32	Management of Immune-Related Adverse Events in Patients Treated With Immune Checkpoint Inhibitor Therapy: American Society of Clinical Oncology Clinical Practice Guideline [32]	ASCO	2018	7	0	0	0	0	0
33	Metastatic Pancreatic Cancer: ASCO Clinical Practice Guideline Update [33]	ASCO	2018	9	11	2	2	0	0
34	Optimizing Anticancer Therapy in Metastatic Non-Castrate Prostate Cancer: American Society of Clinical Oncology Clinical Practice Guideline [34]	ASCO	2018	0	7	0	0	1	0
35	Outpatient Management of Fever and Neutropenia in Adults Treated for Malignancy: American Society of Clinical Oncology and Infectious Diseases Society of America Clinical Practice Guideline Update [35]	ASCO	2018	5	4	1	1	0	0
36	Palliative Care in the Global Setting: ASCO Resource-Stratified Practice Guideline [36]	ASCO	2018	12	6	0	0	0	0
37	Practical Assessment and Management of Vulnerabilities in Older Patients Receiving Chemotherapy: ASCO Guideline for Geriatric Oncology Summary [37]	ASCO	2018	2	2	1	1	0	0
38	Recommendations on Disease Management for Patients With Advanced Human Epidermal Growth Factor Receptor 2–Positive Breast Cancer and Brain Metastases: ASCO Clinical Practice Guideline Update [38]	ASCO	2018	22	0	1	1	0	0
39	Selection of Optimal Adjuvant Chemotherapy and Targeted Therapy for Early Breast Cancer: ASCO Clinical Practice Guideline Focused Update [39]	ASCO	2018	0	3	0	0	0	0

40	Sentinel Lymph Node Biopsy and Management of Regional Lymph Nodes in Melanoma: American Society of Clinical Oncology and Society of Surgical Oncology Clinical Practice Guideline Update [40]	ASCO	2018	0	4	0	0	0	0
41	Systemic Therapy for Patients With Advanced Human Epidermal Growth Factor Receptor 2–Positive Breast Cancer: ASCO Clinical Practice Guideline Update [41]	ASCO	2018	4	12	0	0	0	0
42	Treatment of Malignant Pleural Mesothelioma: American Society of Clinical Oncology Clinical Practice Guideline [42]	ASCO	2018	4	59	1	1	2	0
43	Use of Larynx-Preservation Strategies in the Treatment of Laryngeal Cancer: American Society of Clinical Oncology Clinical Practice Guideline Update [43]	ASCO	2018	0	12	0	0	0	0
44	Adjuvant Systemic Therapy and Adjuvant Radiation Therapy for Stage I to IIIA Completely Resected Non-Small-Cell Lung Cancers: American Society of Clinical Oncology/Cancer Care Ontario Clinical Practice Guideline Update [44]	ASCO	2017	0	5	0	0	0	0
45	Antiemetics: American Society of Clinical Oncology Clinical Practice Guideline Update [45]	ASCO	2017	13	15	2	1	0	0
46	Guideline for the Management of Fever and Neutropenia in Children With Cancer and Hematopoietic Stem-Cell Transplantation Recipients: 2017 Update [46]	ASCO	2017	0	23	0	0	8	2
47	Management of Small Renal Masses: American Society of Clinical Oncology Clinical Practice Guideline [47]	ASCO	2017	0	6	0	0	0	0
48	Molecular Biomarkers for the Evaluation of Colorectal Cancer: Guideline From the American Society for Clinical Pathology, College of American Pathologists, Association for Molecular Pathology, and the American Society of Clinical Oncology [48]	ASCO	2017	11	11	9	5	3	0
49	Patient-Clinician Communication: American Society of Clinical Oncology Consensus Guideline Summary [49]	ASCO	2017	27	3	27	27	0	0
50	Platelet Transfusion for Patients With Cancer: American Society of Clinical Oncology Clinical Practice Guideline Update Summary [50]	ASCO	2017	3	8	0	0	0	0

51	Prevention and Monitoring of Cardiac Dysfunction in Survivors of Adult Cancers: American Society of Clinical Oncology Clinical Practice Guideline [51]	ASCO	2017	8	17	6	4	0	0
52	Primary Prevention of Cervical Cancer: American Society of Clinical Oncology Resource-Stratified Guideline [52]	ASCO	2017	1	23	0	0	0	0
53	Radiation Therapy for Oropharyngeal Squamous Cell Carcinoma: American Society of Clinical Oncology Endorsement of the American Society for Radiation Oncology Evidence-Based Clinical Practice Guideline [53]	ASCO	2017	0	34	0	0	4	0
54	Role of Bone-Modifying Agents in Metastatic Breast Cancer: An American Society of Clinical Oncology–Cancer Care Ontario Focused Guideline Update [54]	ASCO	2017	0	2	0	0	0	0
55	Systemic Therapy for Stage IV Non-Small-Cell Lung Cancer: American Society of Clinical Oncology Clinical Practice Guideline Update [55]	ASCO	2017	9	20	3	2	0	0
56	Treatment of Non-Metastatic Muscle-Invasive Bladder Cancer: AUA/ASCO/ASTRO/SUO Guideline [56]	ASCO	2017	0	14	0	0	4	2
57	Endocrine Therapy for Hormone Receptor–Positive Metastatic Breast Cancer: American Society of Clinical Oncology Guideline [57]	ASCO	2016	2	16	0	0	0	0
58	HER2 Testing and Clinical Decision Making in Gastroesophageal Adenocarcinoma: Guideline From the College of American Pathologists, American Society for Clinical Pathology, and the American Society of Clinical Oncology [58]	ASCO	2016	0	10	0	0	0	0
59	Integration of Palliative Care Into Standard Oncology Care: American Society of Clinical Oncology Clinical Practice Guideline Update [59]	ASCO	2016	1	4	0	0	0	0
60	Locally Advanced, Unresectable Pancreatic Cancer: American Society of Clinical Oncology Clinical Practice Guideline [60]	ASCO	2016	4	13	2	2	0	0
61	Management of Chronic Pain in Survivors of Adult Cancers: American Society of Clinical Oncology Clinical Practice Guideline [61]	ASCO	2016	11	10	1	1	0	0

62	Neoadjuvant Chemotherapy for Newly Diagnosed, Advanced Ovarian Cancer: Society of Gynecologic Oncology and American Society of Clinical Oncology Clinical Practice Guideline [62]	ASCO	2016	4	7	0	0	0	0
63	Postmastectomy Radiotherapy: An American Society of Clinical Oncology, American Society for Radiation Oncology, and Society of Surgical Oncology Focused Guideline Update [63]	ASCO	2016	5	1	2	2	0	0
64	Sentinel Lymph Node Biopsy for Patients With Early-Stage Breast Cancer: American Society of Clinical Oncology Clinical Practice Guideline Update [64]	ASCO	2016	5	6	1	1	0	0
65	Recommendations for the Use of WBC Growth Factors: American Society of Clinical Oncology Clinical Practice Guideline Update [65]	ASCO	2015	2	12	0	0	0	0
66	Use of Biomarkers to Guide Decisions on Systemic Therapy for Women With Metastatic Breast Cancer: American Society of Clinical Oncology Clinical Practice Guideline [66]	ASCO	2015	1	4	0	0	0	0
67	Chemotherapy and Targeted Therapy for Women With Human Epidermal Growth Factor Receptor 2–Negative (or unknown) Advanced Breast Cancer: American Society of Clinical Oncology Clinical Practice Guideline [67]	ASCO	2014	3	6	0	0	0	0
68	Systemic Therapy in Men With Metastatic Castration-Resistant Prostate Cancer: American Society of Clinical Oncology and Cancer Care Ontario Clinical Practice Guideline [68]	ASCO	2014	0	15	0	0	0	0
69	Screening for Prostate Cancer With Prostate-Specific Antigen Testing: American Society of Clinical Oncology Provisional Clinical Opinion [69]	ASCO	2012	1	2	0	0	0	0
70	2020 ACC/AHA Guideline for the Management of Patients With Valvular Heart Disease: A Report of the American College of Cardiology/American Heart Association Joint Committee on Clinical Practice Guidelines [70]	AHA	2021	41	231	28	26	19	17
71	2020 AHA/ACC Guideline for the Diagnosis and Treatment of Patients With Hypertrophic Cardiomyopathy: A Report of the	AHA	2020	16	116	4	4	21	19

	American College of Cardiology/American Heart Association Joint Committee on Clinical Practice Guidelines [71]								
72	2018 ACC/AHA/HRS Guideline on the Evaluation and Management of Patients With Bradycardia and Cardiac Conduction Delay: A Report of the American College of Cardiology/American Heart Association Task Force on Clinical Practice Guidelines and the Heart Rhythm Society [72]	AHA	2019	16	123	5	5	21	11
73	2018 AHA/ACC Guideline for the Management of Adults With Congenital Heart Disease: A Report of the American College of Cardiology/American Heart Association Task Force on Clinical Practice Guidelines [73]	AHA	2019	40	139	22	21	26	22
74	2018 AHA/ACC/AACVPR/AAPA/ABC/ACPM/ADA/AGS/APhA/ASPC/NLA/PCNA Guideline on the Management of Blood Cholesterol: A Report of the American College of Cardiology/American Heart Association Task Force on Clinical Practice Guidelines [74]	AHA	2019	0	74	0	0	3	3
75	2019 ACC/AHA Guideline on the Primary Prevention of Cardiovascular Disease: A Report of the American College of Cardiology/American Heart Association Task Force on Clinical Practice Guidelines [75]	AHA	2019	6	48	5	5	2	1
76	2019 AHA/ACC/HRS Focused Update of the 2014 AHA/ACC/HRS Guideline for the Management of Patients With Atrial Fibrillation: A Report of the American College of Cardiology/American Heart Association Task Force on Clinical Practice Guidelines and the Heart Rhythm Society [76]	AHA	2019	3	35	2	2	0	0
77	2017 ACC/AHA/AAPA/ABC/ACPM/AGS/APhA/ASH/ASPC/NMA/PCNA Guideline for the Prevention, Detection, Evaluation, and Management of High Blood Pressure in Adults: A Report of the American College of Cardiology/American Heart Association Task Force on Clinical Practice Guidelines [77]	AHA	2018	34	81	21	21	5	5
78	2017 AHA/ACC/HRS Guideline for Management of Patients With Ventricular Arrhythmias and the Prevention of Sudden Cardiac	AHA	2018	13	171	7	7	6	3

	Death: A Report of the American College of Cardiology/American Heart Association Task Force on Clinical Practice Guidelines and the Heart Rhythm Society [78]								
79	2016 AHA/ACC Guideline on the Management of Patients With Lower Extremity Peripheral Artery Disease: Executive Summary: A Report of the American College of Cardiology/American Heart Association Task Force on Clinical Practice Guidelines [79]	AHA	2017	14	74	9	8	9	9
80	2017 ACC/AHA/HRS Guideline for the Evaluation and Management of Patients With Syncope: Executive Summary: A Report of the American College of Cardiology/American Heart Association Task Force on Clinical Practice Guidelines and the Heart Rhythm Society [80]	AHA	2017	17	86	12	12	5	5
81	2012 ACCF/AHA/HRS Focused Update Incorporated Into the ACCF/AHA/HRS 2008 Guidelines for Device-Based Therapy of Cardiac Rhythm Abnormalities: A Report of the American College of Cardiology Foundation/American Heart Association Task Force on Practice Guidelines and the Heart Rhythm Society [81]	AHA	2013	0	56	0	0	0	0

Appendix 5: Data analysis codes in STATA/SE 15.1

1. For combined (ACC/AHA + ASCO guidelines)

    1.1 discordant recommendations

    keep if quality_of_evidence==1

    melogit discordant_recommendations type_of_guideline || guideline_ID:,or

```
Mixed-effects logistic regression               Number of obs     =        908
Group variable:     guideline_ID                Number of groups  =         71

                                                Obs per group:
                                                              min =          1
                                                              avg =       12.8
                                                              max =         91

Integration method: mvaghermite                 Integration pts.  =          7

                                                Wald chi2(1)      =      13.18
Log likelihood = -539.95966                     Prob > chi2       =     0.0003
```

discordant_recommendations	Odds Ratio	Std. Err.	z	P>|z|	[95% Conf.	Interval]
type_of_guideline	1.925739	.3476044	3.63	0.000	1.351923	2.743109
_cons	.213981	.0629811	-5.24	0.000	.1201824	.3809865
**guideline_ID**						
var(_cons)	2.896993	.9961553			1.476572	5.683819

```
Note: Estimates are transformed only in the first equation.
Note: _cons estimates baseline odds (conditional on zero random effects).
LR test vs. logistic model: chibar2(01) = 130.11       Prob >= chibar2 = 0.0000
```

    1.2 inappropriate discordant recommendations

    melogit inappropriate_discordant_recom type_of_guideline || guideline_ID:,or

```
Mixed-effects logistic regression               Number of obs     =        908
Group variable:     guideline_ID                Number of groups  =         71

                                                Obs per group:
                                                              min =          1
                                                              avg =       12.8
                                                              max =         91

Integration method: mvaghermite                 Integration pts.  =          7

                                                Wald chi2(1)      =      23.35
Log likelihood = -488.19328                     Prob > chi2       =     0.0000
```

inappropriate_discordant_recom	Odds Ratio	Std. Err.	z	P>|z|	[95% Conf.	Interval]
type_of_guideline	2.457253	.4572262	4.83	0.000	1.706338	3.538627
_cons	.1113845	.0334806	-7.30	0.000	.0617968	.200763
**guideline_ID**						
var(_cons)	2.600905	.892456			1.327545	5.095652

```
Note: Estimates are transformed only in the first equation.
Note: _cons estimates baseline odds (conditional on zero random effects).
LR test vs. logistic model: chibar2(01) = 143.09       Prob >= chibar2 = 0.0000
```

2. ACC/AHA guidelines

    2.1 discordant recommendations

    drop if organization=="ASCO"

    keep if quality_of_evidence==1

    melogit discordant_recommendations type_of_guideline || guideline_ID:,or

```
Mixed-effects logistic regression               Number of obs     =        504
Group variable:     guideline_ID                Number of groups  =         11

                                                Obs per group:
                                                              min =          4
                                                              avg =       45.8
                                                              max =         91

Integration method: mvaghermite                 Integration pts.  =          7

                                                Wald chi2(1)      =      15.58
Log likelihood = -338.88962                     Prob > chi2       =     0.0001
```

discordant_recommendations	Odds Ratio	Std. Err.	z	P>|z|	[95% Conf.	Interval]
type_of_guideline	2.128236	.4072979	3.95	0.000	1.462575	3.096857
_cons	.6263696	.0787612	-3.72	0.000	.4895517	.8014248
**guideline_ID**						
var(_cons)	.0141588	.0447583			.0000289	6.947325

```
Note: Estimates are transformed only in the first equation.
Note: _cons estimates baseline odds (conditional on zero random effects).
LR test vs. logistic model: chibar2(01) = 0.13         Prob >= chibar2 = 0.3581
```

2.2 inappropriate discordant recommendations

melogit discordant_recommendations type_of_guideline || guideline_ID:,or

```
Mixed-effects logistic regression               Number of obs     =        504
Group variable:     guideline_ID                Number of groups  =         11

                                                Obs per group:
                                                              min =          4
                                                              avg =       45.8
                                                              max =         91

Integration method: mvaghermite                 Integration pts.  =          7

                                                Wald chi2(1)      =      22.84
Log likelihood = -324.83895                     Prob > chi2       =     0.0000
------------------------------------------------------------------------------
inappropriate_discordant_recom | Odds Ratio   Std. Err.      z    P>|z|     [95% Conf. Interval]
-------------------------------+----------------------------------------------------------------
             type_of_guideline |    2.55646   .5021438     4.78   0.000     1.739581    3.756932
                         _cons |    .464328    .074495    -4.78   0.000     .3390481    .6358994
-------------------------------+----------------------------------------------------------------
guideline_ID                   |
                     var(_cons)|   .0837928   .0826785                         .012115    .5795467
------------------------------------------------------------------------------------------------
Note: Estimates are transformed only in the first equation.
Note: _cons estimates baseline odds (conditional on zero random effects).
LR test vs. logistic model: chibar2(01) = 2.78         Prob >= chibar2 = 0.0478
```

3. ASCO guidelines

3.1 discordant recommendations

drop if organization=="AHA"

keep if quality_of_evidence==1

melogit discordant_recommendations type_of_guideline || guideline_ID:,or

```
Mixed-effects logistic regression               Number of obs     =        404
Group variable:     guideline_ID                Number of groups  =         60

                                                Obs per group:
                                                              min =          1
                                                              avg =        6.7
                                                              max =         38

Integration method: mvaghermite                 Integration pts.  =          7

                                                Wald chi2(1)      =       4.56
Log likelihood = -186.51493                     Prob > chi2       =     0.0328
------------------------------------------------------------------------------
discordant_recommendations | Odds Ratio   Std. Err.      z    P>|z|     [95% Conf. Interval]
---------------------------+----------------------------------------------------------------
         type_of_guideline |   2.928578   1.473984     2.13   0.033     1.092039    7.853718
                     _cons |   .0963386   .0530134    -4.25   0.000     .0327642    .2832704
---------------------------+----------------------------------------------------------------
guideline_ID               |
                 var(_cons)|   5.102848    2.10434                        2.273995     11.4508
--------------------------------------------------------------------------------------------
Note: Estimates are transformed only in the first equation.
Note: _cons estimates baseline odds (conditional on zero random effects).
LR test vs. logistic model: chibar2(01) = 121.03       Prob >= chibar2 = 0.0000
```

3.2 inappropriate discordant recommendations

melogit discordant_recommendations type_of_guideline || guideline_ID:,or

```
Mixed-effects logistic regression               Number of obs     =        404
Group variable:     guideline_ID                Number of groups  =         60

                                                Obs per group:
                                                              min =          1
                                                              avg =        6.7
                                                              max =         38

Integration method: mvaghermite                 Integration pts.  =          7

                                                Wald chi2(1)      =       7.67
Log likelihood = -148.36165                     Prob > chi2       =     0.0056
------------------------------------------------------------------------------
inappropriate_discordant_recom | Odds Ratio   Std. Err.      z    P>|z|     [95% Conf. Interval]
-------------------------------+----------------------------------------------
             type_of_guideline |   5.065468   2.966848    2.77   0.006     1.607201      15.965
                         _cons |   .0357963   .0217146   -5.49   0.000     .0109015    .1175414
-------------------------------+----------------------------------------------
guideline_ID                   |
                     var(_cons)|   3.795157   1.636761                      1.629772    8.837566
------------------------------------------------------------------------------
Note: Estimates are transformed only in the first equation.
Note: _cons estimates baseline odds (conditional on zero random effects).
LR test vs. logistic model: chibar2(01) = 96.86       Prob >= chibar2 = 0.0000
```

Appendix 6. Proportion of recommendations with "against" vs "favor" interventions in the discordant recommendations and inappropriate discordant recommendations*

Measurement	Type of recommendation	ACC/AHA		ASCO	
		Against intervention	Favor intervention	Against intervention	Favor intervention
Discordant recommendations	Consensus	2 (2%)	113 (98%)	16 (17%)	76 (83%)
	Evidence	22 (19%)	95 (81%)	12 (40%)	18 (60%)
Inappropriate discordant recommendations	Consensus	0 (0%)	111 (100%)	15 (21%)	57 (79 %)
	Evidence	1 (1%)	94 (99%)	5 (50%)	5 (50%)

* Because of the small number of inappropriate discordant recommendations, we did not perform formal statistical analysis.

Appendix 7 : References of included guidelines

1. Virgo KS, Rumble RB, de Wit R, et al. Initial Management of Noncastrate Advanced, Recurrent, or Metastatic Prostate Cancer: ASCO Guideline Update. *Journal of clinical oncology : official journal of the American Society of Clinical Oncology* 2021;39(11):1274-305. doi: 10.1200/jco.20.03256 [published Online First: 2021/01/27]
2. Hui D, Bohlke K, Bao T, et al. Management of Dyspnea in Advanced Cancer: ASCO Guideline. *Journal of clinical oncology : official journal of the American Society of Clinical Oncology* 2021;39(12):1389-411. doi: 10.1200/jco.20.03465 [published Online First: 2021/02/23]
3. Korde LA, Somerfield MR, Carey LA, et al. Neoadjuvant Chemotherapy, Endocrine Therapy, and Targeted Therapy for Breast Cancer: ASCO Guideline. *Journal of clinical oncology : official journal of the American Society of Clinical Oncology* 2021;39(13):1485-505. doi: 10.1200/jco.20.03399 [published Online First: 2021/01/29]
4. Hanna NH, Robinson AG, Temin S, et al. Therapy for Stage IV Non-Small-Cell Lung Cancer With Driver Alterations: ASCO and OH (CCO) Joint Guideline Update. *Journal of clinical oncology : official journal of the American Society of Clinical Oncology* 2021;39(9):1040-91. doi: 10.1200/jco.20.03570 [published Online First: 2021/02/17]
5. Maghami E, Ismaila N, Alvarez A, et al. Diagnosis and Management of Squamous Cell Carcinoma of Unknown Primary in the Head and Neck: ASCO Guideline. *Journal of clinical oncology : official journal of the American Society of Clinical Oncology* 2020;38(22):2570-96. doi: 10.1200/jco.20.00275 [published Online First: 2020/04/24]

6. Allison KH, Hammond MEH, Dowsett M, et al. Estrogen and Progesterone Receptor Testing in Breast Cancer: ASCO/CAP Guideline Update. *Journal of clinical oncology : official journal of the American Society of Clinical Oncology* 2020;38(12):1346-66. doi: 10.1200/jco.19.02309 [published Online First: 2020/01/14]
7. Konstantinopoulos PA, Norquist B, Lacchetti C, et al. Germline and Somatic Tumor Testing in Epithelial Ovarian Cancer: ASCO Guideline. *Journal of clinical oncology : official journal of the American Society of Clinical Oncology* 2020;38(11):1222-45. doi: 10.1200/jco.19.02960 [published Online First: 2020/01/28]
8. Schneider BJ, Ismaila N, Aerts J, et al. Lung Cancer Surveillance After Definitive Curative-Intent Therapy: ASCO Guideline. *Journal of clinical oncology : official journal of the American Society of Clinical Oncology* 2020;38(7):753-66. doi: 10.1200/jco.19.02748 [published Online First: 2019/12/13]
9. Roeland EJ, Bohlke K, Baracos VE, et al. Management of Cancer Cachexia: ASCO Guideline. *Journal of clinical oncology : official journal of the American Society of Clinical Oncology* 2020;38(21):2438-53. doi: 10.1200/jco.20.00611 [published Online First: 2020/05/21]
10. Tung NM, Boughey JC, Pierce LJ, et al. Management of Hereditary Breast Cancer: American Society of Clinical Oncology, American Society for Radiation Oncology, and Society of Surgical Oncology Guideline. *Journal of clinical oncology : official journal of the American Society of Clinical Oncology* 2020;38(18):2080-106. doi: 10.1200/jco.20.00299 [published Online First: 2020/04/04]
11. Hassett MJ, Somerfield MR, Baker ER, et al. Management of Male Breast Cancer: ASCO Guideline. *Journal of clinical oncology : official journal of the American Society of Clinical Oncology* 2020;38(16):1849-63. doi: 10.1200/jco.19.03120 [published Online First: 2020/02/15]
12. Eggener SE, Rumble RB, Armstrong AJ, et al. Molecular Biomarkers in Localized Prostate Cancer: ASCO Guideline. *Journal of clinical oncology : official journal of the American Society of Clinical Oncology* 2020;38(13):1474-94. doi: 10.1200/jco.19.02768 [published Online First: 2019/12/13]
13. Trabulsi EJ, Rumble RB, Jadvar H, et al. Optimum Imaging Strategies for Advanced Prostate Cancer: ASCO Guideline. *Journal of clinical oncology : official journal of the American Society of Clinical Oncology* 2020;38(17):1963-96. doi: 10.1200/jco.19.02757 [published Online First: 2020/01/16]
14. Loprinzi CL, Lacchetti C, Bleeker J, et al. Prevention and Management of Chemotherapy-Induced Peripheral Neuropathy in Survivors of Adult Cancers: ASCO Guideline Update. *Journal of Clinical Oncology* 2020;38(28):3325-48. doi: 10.1200/JCO.20.01399
15. Seth R, Messersmith H, Kaur V, et al. Systemic Therapy for Melanoma: ASCO Guideline. *Journal of clinical oncology : official journal of the American Society of Clinical Oncology* 2020;38(33):3947-70. doi: 10.1200/jco.20.00198 [published Online First: 2020/04/02]
16. Hanna NH, Schneider BJ, Temin S, et al. Therapy for Stage IV Non-Small-Cell Lung Cancer Without Driver Alterations: ASCO and OH (CCO) Joint Guideline Update. *Journal of clinical oncology : official journal of the American Society of Clinical Oncology* 2020;38(14):1608-32. doi: 10.1200/jco.19.03022 [published Online First: 2020/01/29]
17. Shroff RT, Kennedy EB, Bachini M, et al. Adjuvant Therapy for Resected Biliary Tract Cancer: ASCO Clinical Practice Guideline. *Journal of Clinical Oncology* 2019;37(12):1015-27. doi: 10.1200/JCO.18.02178
18. Lieu C, Kennedy EB, Bergsland E, et al. Duration of Oxaliplatin-Containing Adjuvant Therapy for Stage III Colon Cancer: ASCO Clinical Practice Guideline. *Journal of Clinical Oncology* 2019;37(16):1436-47. doi: 10.1200/JCO.19.00281
19. Bohlius J, Bohlke K, Castelli R, et al. Management of Cancer-Associated Anemia With Erythropoiesis-Stimulating Agents: ASCO/ASH Clinical Practice Guideline Update. *Journal of Clinical Oncology* 2019;37(15):1336-51. doi: 10.1200/JCO.18.02142
20. Shapiro CL, Van Poznak C, Lacchetti C, et al. Management of Osteoporosis in Survivors of Adult Cancers With Nonmetastatic Disease: ASCO Clinical Practice Guideline. *Journal of clinical oncology : official journal of the American Society of Clinical Oncology* 2019;37(31):2916-46. doi: 10.1200/jco.19.01696 [published Online First: 2019/09/19]
21. Koyfman SA, Ismaila N, Crook D, et al. Management of the Neck in Squamous Cell Carcinoma of the Oral Cavity and Oropharynx: ASCO Clinical Practice Guideline. *Journal of clinical oncology : official journal of the American Society of Clinical Oncology* 2019;37(20):1753-74. doi: 10.1200/jco.18.01921 [published Online First: 2019/02/28]
22. Yarom N, Shapiro CL, Peterson DE, et al. Medication-Related Osteonecrosis of the Jaw: MASCC/ISOO/ASCO Clinical Practice Guideline. *Journal of Clinical Oncology* 2019;37(25):2270-90. doi: 10.1200/JCO.19.01186
23. Khorana AA, McKernin SE, Berlin J, et al. Potentially Curable Pancreatic Adenocarcinoma: ASCO Clinical Practice Guideline Update. *Journal of Clinical Oncology* 2019;37(23):2082-88. doi: 10.1200/JCO.19.00946

24. Henry NL, Somerfield MR, Abramson VG, et al. Role of Patient and Disease Factors in Adjuvant Systemic Therapy Decision Making for Early-Stage, Operable Breast Cancer: Update of the ASCO Endorsement of the Cancer Care Ontario Guideline. *Journal of Clinical Oncology* 2019;37(22):1965-77. doi: 10.1200/JCO.19.00948
25. Mikhael J, Ismaila N, Cheung MC, et al. Treatment of Multiple Myeloma: ASCO and CCO Joint Clinical Practice Guideline. *Journal of clinical oncology : official journal of the American Society of Clinical Oncology* 2019;37(14):1228-63. doi: 10.1200/jco.18.02096 [published Online First: 2019/04/02]
26. Andre F, Ismaila N, Henry NL, et al. Use of Biomarkers to Guide Decisions on Adjuvant Systemic Therapy for Women With Early-Stage Invasive Breast Cancer: ASCO Clinical Practice Guideline Update—Integration of Results From TAILORx. *Journal of Clinical Oncology* 2019;37(22):1956-64. doi: 10.1200/JCO.19.00945
27. Visvanathan K, Fabian CJ, Bantug E, et al. Use of Endocrine Therapy for Breast Cancer Risk Reduction: ASCO Clinical Practice Guideline Update. *Journal of Clinical Oncology* 2019;37(33):3152-65. doi: 10.1200/JCO.19.01472
28. Key NS, Khorana AA, Kuderer NM, et al. Venous Thromboembolism Prophylaxis and Treatment in Patients With Cancer: ASCO Clinical Practice Guideline Update. *Journal of Clinical Oncology* 2019;38(5):496-520. doi: 10.1200/JCO.19.01461
29. Taplitz RA, Kennedy EB, Flowers CR. Antimicrobial Prophylaxis for Adult Patients With Cancer-Related Immunosuppression: ASCO and IDSA Clinical Practice Guideline Update Summary. *Journal of Oncology Practice* 2018;14(11):692-95. doi: 10.1200/JOP.18.00366
30. Stoffel EM, McKernin SE, Brand R, et al. Evaluating Susceptibility to Pancreatic Cancer: ASCO Provisional Clinical Opinion. *Journal of Clinical Oncology* 2018;37(2):153-64. doi: 10.1200/JCO.18.01489
31. Morgan SC, Hoffman K, Loblaw DA, et al. Hypofractionated Radiation Therapy for Localized Prostate Cancer: An ASTRO, ASCO, and AUA Evidence-Based Guideline. *Journal of Clinical Oncology* 2018;36(34):3411-30. doi: 10.1200/JCO.18.01097
32. Brahmer JR, Lacchetti C, Schneider BJ, et al. Management of Immune-Related Adverse Events in Patients Treated With Immune Checkpoint Inhibitor Therapy: American Society of Clinical Oncology Clinical Practice Guideline. *Journal of Clinical Oncology* 2018;36(17):1714-68. doi: 10.1200/JCO.2017.77.6385
33. Sohal DPS, Kennedy EB, Khorana A, et al. Metastatic Pancreatic Cancer: ASCO Clinical Practice Guideline Update. *Journal of Clinical Oncology* 2018;36(24):2545-56. doi: 10.1200/JCO.2018.78.9636
34. Morris MJ, Rumble RB, Basch E, et al. Optimizing Anticancer Therapy in Metastatic Non-Castrate Prostate Cancer: American Society of Clinical Oncology Clinical Practice Guideline. *Journal of Clinical Oncology* 2018;36(15):1521-39. doi: 10.1200/JCO.2018.78.0619
35. Taplitz RA, Kennedy EB, Bow EJ, et al. Outpatient Management of Fever and Neutropenia in Adults Treated for Malignancy: American Society of Clinical Oncology and Infectious Diseases Society of America Clinical Practice Guideline Update. *Journal of Clinical Oncology* 2018;36(14):1443-53. doi: 10.1200/JCO.2017.77.6211
36. Osman H, Shrestha S, Temin S, et al. Palliative Care in the Global Setting: ASCO Resource-Stratified Practice Guideline. *Journal of global oncology* 2018;4:1-24. doi: 10.1200/jgo.18.00026 [published Online First: 2018/08/08]
37. Mohile SG, Dale W, Somerfield MR, et al. Practical Assessment and Management of Vulnerabilities in Older Patients Receiving Chemotherapy: ASCO Guideline for Geriatric Oncology Summary. *Journal of Oncology Practice* 2018;14(7):442-46. doi: 10.1200/JOP.18.00180
38. Ramakrishna N, Temin S, Chandarlapaty S, et al. Recommendations on Disease Management for Patients With Advanced Human Epidermal Growth Factor Receptor 2–Positive Breast Cancer and Brain Metastases: ASCO Clinical Practice Guideline Update. *Journal of Clinical Oncology* 2018;36(27):2804-07. doi: 10.1200/JCO.2018.79.2713
39. Denduluri N, Chavez-MacGregor M, Telli ML, et al. Selection of Optimal Adjuvant Chemotherapy and Targeted Therapy for Early Breast Cancer: ASCO Clinical Practice Guideline Focused Update. *Journal of Clinical Oncology* 2018;36(23):2433-43. doi: 10.1200/JCO.2018.78.8604
40. Wong SL, Faries MB, Kennedy EB, et al. Sentinel Lymph Node Biopsy and Management of Regional Lymph Nodes in Melanoma: American Society of Clinical Oncology and Society of Surgical Oncology Clinical Practice Guideline Update. *Journal of clinical oncology : official journal of the American Society of Clinical Oncology* 2018;36(4):399-413. doi: 10.1200/jco.2017.75.7724 [published Online First: 2017/12/13]
41. Giordano SH, Temin S, Chandarlapaty S, et al. Systemic Therapy for Patients With Advanced Human Epidermal Growth Factor Receptor 2–Positive Breast Cancer: ASCO Clinical Practice Guideline Update. *Journal of Clinical Oncology* 2018;36(26):2736-40. doi: 10.1200/JCO.2018.79.2697

42. Kindler HL, Ismaila N, Armato SG, 3rd, et al. Treatment of Malignant Pleural Mesothelioma: American Society of Clinical Oncology Clinical Practice Guideline. *Journal of clinical oncology : official journal of the American Society of Clinical Oncology* 2018;36(13):1343-73. doi: 10.1200/jco.2017.76.6394 [published Online First: 2018/01/19]
43. Forastiere AA, Ismaila N, Lewin JS, et al. Use of Larynx-Preservation Strategies in the Treatment of Laryngeal Cancer: American Society of Clinical Oncology Clinical Practice Guideline Update. *Journal of clinical oncology : official journal of the American Society of Clinical Oncology* 2018;36(11):1143-69. doi: 10.1200/jco.2017.75.7385 [published Online First: 2017/11/28]
44. Kris MG, Gaspar LE, Chaft JE, et al. Adjuvant Systemic Therapy and Adjuvant Radiation Therapy for Stage I to IIIA Completely Resected Non-Small-Cell Lung Cancers: American Society of Clinical Oncology/Cancer Care Ontario Clinical Practice Guideline Update. *Journal of clinical oncology : official journal of the American Society of Clinical Oncology* 2017;35(25):2960-74. doi: 10.1200/jco.2017.72.4401 [published Online First: 2017/04/25]
45. Hesketh PJ, Kris MG, Basch E, et al. Antiemetics: American Society of Clinical Oncology Clinical Practice Guideline Update. *Journal of Clinical Oncology* 2017;35(28):3240-61. doi: 10.1200/JCO.2017.74.4789
46. Lehrnbecher T, Robinson P, Fisher B, et al. Guideline for the Management of Fever and Neutropenia in Children With Cancer and Hematopoietic Stem-Cell Transplantation Recipients: 2017 Update. *Journal of Clinical Oncology* 2017;35(18):2082-94. doi: 10.1200/JCO.2016.71.7017
47. Finelli A, Ismaila N, Bro B, et al. Management of Small Renal Masses: American Society of Clinical Oncology Clinical Practice Guideline. *Journal of Clinical Oncology* 2017;35(6):668-80. doi: 10.1200/JCO.2016.69.9645
48. Sepulveda AR, Hamilton SR, Allegra CJ, et al. Molecular Biomarkers for the Evaluation of Colorectal Cancer: Guideline From the American Society for Clinical Pathology, College of American Pathologists, Association for Molecular Pathology, and the American Society of Clinical Oncology. *Journal of Clinical Oncology* 2017;35(13):1453-86. doi: 10.1200/JCO.2016.71.9807
49. Gilligan T, Bohlke K, Baile WF. Patient-Clinician Communication: American Society of Clinical Oncology Consensus Guideline Summary. *Journal of Oncology Practice* 2017;14(1):42-46. doi: 10.1200/JOP.2017.027144
50. Schiffer CA, Bohlke K, Anderson KC. Platelet Transfusion for Patients With Cancer: American Society of Clinical Oncology Clinical Practice Guideline Update Summary. *Journal of Oncology Practice* 2017;14(2):129-33. doi: 10.1200/JOP.2017.028902
51. Armenian SH, Lacchetti C, Barac A, et al. Prevention and Monitoring of Cardiac Dysfunction in Survivors of Adult Cancers: American Society of Clinical Oncology Clinical Practice Guideline. *Journal of clinical oncology : official journal of the American Society of Clinical Oncology* 2017;35(8):893-911. doi: 10.1200/jco.2016.70.5400 [published Online First: 2016/12/06]
52. Arrossi S, Temin S, Garland S, et al. Primary Prevention of Cervical Cancer: American Society of Clinical Oncology Resource-Stratified Guideline. *Journal of global oncology* 2017;3(5):611-34. doi: 10.1200/jgo.2016.008151 [published Online First: 2017/11/03]
53. Quon H, Vapiwala N, Forastiere A, et al. Radiation Therapy for Oropharyngeal Squamous Cell Carcinoma: American Society of Clinical Oncology Endorsement of the American Society for Radiation Oncology Evidence-Based Clinical Practice Guideline. *Journal of clinical oncology : official journal of the American Society of Clinical Oncology* 2017;35(36):4078-90. doi: 10.1200/jco.2017.73.8633 [published Online First: 2017/10/25]
54. Van Poznak C, Somerfield MR, Barlow WE, et al. Role of Bone-Modifying Agents in Metastatic Breast Cancer: An American Society of Clinical Oncology–Cancer Care Ontario Focused Guideline Update. *Journal of Clinical Oncology* 2017;35(35):3978-86. doi: 10.1200/JCO.2017.75.4614
55. Hanna N, Johnson D, Temin S, et al. Systemic Therapy for Stage IV Non-Small-Cell Lung Cancer: American Society of Clinical Oncology Clinical Practice Guideline Update. *Journal of clinical oncology : official journal of the American Society of Clinical Oncology* 2017;35(30):3484-515. doi: 10.1200/jco.2017.74.6065 [published Online First: 2017/08/15]
56. Chang SS, Bochner BH, Chou R, et al. Treatment of Non-Metastatic Muscle-Invasive Bladder Cancer: AUA/ASCO/ASTRO/SUO Guideline. *The Journal of urology* 2017;198(3):552-59. doi: 10.1016/j.juro.2017.04.086 [published Online First: 2017/05/01]
57. Rugo HS, Rumble RB, Macrae E, et al. Endocrine Therapy for Hormone Receptor–Positive Metastatic Breast Cancer: American Society of Clinical Oncology Guideline. *Journal of Clinical Oncology* 2016;34(25):3069-103. doi: 10.1200/JCO.2016.67.1487
58. Bartley AN, Washington MK, Colasacco C, et al. HER2 Testing and Clinical Decision Making in

Gastroesophageal Adenocarcinoma: Guideline From the College of American Pathologists, American Society for Clinical Pathology, and the American Society of Clinical Oncology. *Journal of Clinical Oncology* 2016;35(4):446-64. doi: 10.1200/JCO.2016.69.4836
59. Ferrell BR, Temel JS, Temin S, et al. Integration of Palliative Care Into Standard Oncology Care: American Society of Clinical Oncology Clinical Practice Guideline Update. *Journal of Clinical Oncology* 2016;35(1):96-112. doi: 10.1200/JCO.2016.70.1474
60. Balaban EP, Mangu PB, Khorana AA, et al. Locally Advanced, Unresectable Pancreatic Cancer: American Society of Clinical Oncology Clinical Practice Guideline. *Journal of Clinical Oncology* 2016;34(22):2654-68. doi: 10.1200/JCO.2016.67.5561
61. Paice JA, Portenoy R, Lacchetti C, et al. Management of Chronic Pain in Survivors of Adult Cancers: American Society of Clinical Oncology Clinical Practice Guideline. *Journal of clinical oncology : official journal of the American Society of Clinical Oncology* 2016;34(27):3325-45. doi: 10.1200/jco.2016.68.5206 [published Online First: 2016/07/28]
62. Wright AA, Bohlke K, Armstrong DK, et al. Neoadjuvant Chemotherapy for Newly Diagnosed, Advanced Ovarian Cancer: Society of Gynecologic Oncology and American Society of Clinical Oncology Clinical Practice Guideline. *Journal of clinical oncology : official journal of the American Society of Clinical Oncology* 2016;34(28):3460-73. doi: 10.1200/jco.2016.68.6907 [published Online First: 2016/08/10]
63. Recht A, Comen EA, Fine RE, et al. Postmastectomy Radiotherapy: An American Society of Clinical Oncology, American Society for Radiation Oncology, and Society of Surgical Oncology Focused Guideline Update. *Journal of Clinical Oncology* 2016;34(36):4431-42. doi: 10.1200/JCO.2016.69.1188
64. Lyman GH, Somerfield MR, Bosserman LD, et al. Sentinel Lymph Node Biopsy for Patients With Early-Stage Breast Cancer: American Society of Clinical Oncology Clinical Practice Guideline Update. *Journal of Clinical Oncology* 2016;35(5):561-64. doi: 10.1200/JCO.2016.71.0947
65. Smith TJ, Bohlke K, Lyman GH, et al. Recommendations for the Use of WBC Growth Factors: American Society of Clinical Oncology Clinical Practice Guideline Update. *Journal of Clinical Oncology* 2015;33(28):3199-212. doi: 10.1200/JCO.2015.62.3488
66. Van Poznak C, Somerfield MR, Bast RC, et al. Use of Biomarkers to Guide Decisions on Systemic Therapy for Women With Metastatic Breast Cancer: American Society of Clinical Oncology Clinical Practice Guideline. *Journal of Clinical Oncology* 2015;33(24):2695-704. doi: 10.1200/JCO.2015.61.1459
67. Partridge AH, Rumble RB, Carey LA, et al. Chemotherapy and Targeted Therapy for Women With Human Epidermal Growth Factor Receptor 2–Negative (or unknown) Advanced Breast Cancer: American Society of Clinical Oncology Clinical Practice Guideline. *Journal of Clinical Oncology* 2014;32(29):3307-29. doi: 10.1200/JCO.2014.56.7479
68. Basch E, Loblaw DA, Oliver TK, et al. Systemic Therapy in Men With Metastatic Castration-Resistant Prostate Cancer: American Society of Clinical Oncology and Cancer Care Ontario Clinical Practice Guideline. *Journal of Clinical Oncology* 2014;32(30):3436-48. doi: 10.1200/JCO.2013.54.8404
69. Basch E, Oliver TK, Vickers A, et al. Screening for Prostate Cancer With Prostate-Specific Antigen Testing: American Society of Clinical Oncology Provisional Clinical Opinion. *Journal of Clinical Oncology* 2012;30(24):3020-25. doi: 10.1200/JCO.2012.43.3441
70. Writing Committee M, Otto CM, Nishimura RA, et al. 2020 ACC/AHA Guideline for the Management of Patients With Valvular Heart Disease: A Report of the American College of Cardiology/American Heart Association Joint Committee on Clinical Practice Guidelines. *J Am Coll Cardiol* 2021;77(4):e25-e197. doi: 10.1016/j.jacc.2020.11.018 [published Online First: 2020/12/22]
71. Ommen SR, Mital S, Burke MA, et al. 2020 AHA/ACC Guideline for the Diagnosis and Treatment of Patients With Hypertrophic Cardiomyopathy: A Report of the American College of Cardiology/American Heart Association Joint Committee on Clinical Practice Guidelines. *J Am Coll Cardiol* 2020;76(25):e159-e240. doi: 10.1016/j.jacc.2020.08.045 [published Online First: 2020/11/25]
72. Kusumoto FM, Schoenfeld MH, Barrett C, et al. 2018 ACC/AHA/HRS Guideline on the Evaluation and Management of Patients With Bradycardia and Cardiac Conduction Delay: A Report of the American College of Cardiology/American Heart Association Task Force on Clinical Practice Guidelines and the Heart Rhythm Society. *J Am Coll Cardiol* 2019;74(7):e51-e156. doi: 10.1016/j.jacc.2018.10.044 [published Online First: 2018/11/10]
73. Stout KK, Daniels CJ, Aboulhosn JA, et al. 2018 AHA/ACC Guideline for the Management of Adults With Congenital Heart Disease: A Report of the American College of Cardiology/American Heart Association Task Force on Clinical Practice Guidelines. *J Am Coll Cardiol* 2019;73(12):e81-e192. doi: 10.1016/j.jacc.2018.08.1029 [published Online First: 2018/08/20]
74. Grundy SM, Stone NJ, Bailey AL, et al. 2018

AHA/ACC/AACVPR/AAPA/ABC/ACPM/ADA/AGS/APhA/ASPC/NLA/PCNA Guideline on the Management of Blood Cholesterol: A Report of the American College of Cardiology/American Heart Association Task Force on Clinical Practice Guidelines. *J Am Coll Cardiol* 2019;73(24):e285-e350. doi: 10.1016/j.jacc.2018.11.003 [published Online First: 2018/11/14]

75. Arnett DK, Blumenthal RS, Albert MA, et al. 2019 ACC/AHA Guideline on the Primary Prevention of Cardiovascular Disease: A Report of the American College of Cardiology/American Heart Association Task Force on Clinical Practice Guidelines. *J Am Coll Cardiol* 2019;74(10):e177-e232. doi: 10.1016/j.jacc.2019.03.010 [published Online First: 2019/03/22]
76. January CT, Wann LS, Calkins H, et al. 2019 AHA/ACC/HRS Focused Update of the 2014 AHA/ACC/HRS Guideline for the Management of Patients With Atrial Fibrillation: A Report of the American College of Cardiology/American Heart Association Task Force on Clinical Practice Guidelines and the Heart Rhythm Society. *J Am Coll Cardiol* 2019;74(1):104-32. doi: 10.1016/j.jacc.2019.01.011 [published Online First: 2019/02/01]
77. Whelton PK, Carey RM, Aronow WS, et al. 2017 ACC/AHA/AAPA/ABC/ACPM/AGS/APhA/ASH/ASPC/NMA/PCNA Guideline for the Prevention, Detection, Evaluation, and Management of High Blood Pressure in Adults: A Report of the American College of Cardiology/American Heart Association Task Force on Clinical Practice Guidelines. *J Am Coll Cardiol* 2018;71(19):e127-e248. doi: 10.1016/j.jacc.2017.11.006 [published Online First: 2017/11/18]
78. Al-Khatib SM, Stevenson WG, Ackerman MJ, et al. 2017 AHA/ACC/HRS Guideline for Management of Patients With Ventricular Arrhythmias and the Prevention of Sudden Cardiac Death: A Report of the American College of Cardiology/American Heart Association Task Force on Clinical Practice Guidelines and the Heart Rhythm Society. *J Am Coll Cardiol* 2018;72(14):e91-e220. doi: 10.1016/j.jacc.2017.10.054 [published Online First: 2017/11/04]
79. Gerhard-Herman MD, Gornik HL, Barrett C, et al. 2016 AHA/ACC Guideline on the Management of Patients With Lower Extremity Peripheral Artery Disease: Executive Summary: A Report of the American College of Cardiology/American Heart Association Task Force on Clinical Practice Guidelines. *Circulation* 2017;135(12):e686-e725. doi: 10.1161/CIR.0000000000000470
80. Shen W-K, Sheldon RS, Benditt DG, et al. 2017 ACC/AHA/HRS Guideline for the Evaluation and Management of Patients With Syncope: Executive Summary: A Report of the American College of Cardiology/American Heart Association Task Force on Clinical Practice Guidelines and the Heart Rhythm Society. *Circulation* 2017;136(5):e25-e59. doi: 10.1161/CIR.0000000000000498
81. Epstein AE, DiMarco JP, Ellenbogen KA, et al. 2012 ACCF/AHA/HRS focused update incorporated into the ACCF/AHA/HRS 2008 guidelines for device-based therapy of cardiac rhythm abnormalities: a report of the American College of Cardiology Foundation/American Heart Association Task Force on Practice Guidelines and the Heart Rhythm Society. *J Am Coll Cardiol* 2013;61(3):e6-75. doi: 10.1016/j.jacc.2012.11.007 [published Online First: 2012/12/26]
